# School and community drivers of child diets in two Arab cities: The SCALE protocol and innovative tools to assess children’s food environments

**DOI:** 10.1371/journal.pone.0264963

**Published:** 2022-07-20

**Authors:** Hala Ghattas, Zeina Jamaluddine, Aline Semaan, Nehmat El-Helou, Gloria Safadi, Tatiana Elghossain, Christelle Akl, Shady Elbassuoni, Ali Chalak, Jalila El Ati, the SCALE Research Group

**Affiliations:** 1 Center for Research on Population and Health, Faculty of Health Sciences, American University of Beirut, Beirut, Lebanon; 2 London School of Hygiene and Tropical Medicine, London, United Kingdom; 3 Department of Public Health, Institute of Tropical Medicine, Antwerp, Belgium; 4 Department of Computer Science, Faculty of Arts and Sciences, American University of Beirut, Beirut, Lebanon; 5 Department of Agriculture, Faculty of Agricultural and Food Sciences, American University of Beirut, Beirut, Lebanon; 6 INNTA (National Institute of Nutrition and Food Technology), SURVEN (Nutrition Surveillance and Epidemiology in Tunisia) Research Laboratory, Tunis, Tunisia; UNICEF India, Government Medical College, INDIA

## Abstract

**Background:**

In the context of the rapid nutrition transition experienced by middle-income countries of the Arab region, children and adolescent’s food choices and dietary behaviors are early risk factors for the development of non-communicable diseases. Assessment of factors influencing food choices among this age group is challenging and is usually based on self-reported data, which are prone to information and recall bias. As the popularity of technologies and video gaming platforms increases, opportunities arise to use these tools to collect data on variables that affect food choice, dietary intake, and associated outcomes. This protocol paper describes the SCALE study (School and community drivers of child diets in Arab cities; identifying levers for intervention) which aims to explore the environments at the level of households, schools and communities in which children’s food choices are made and consequently identify barriers and enablers to healthy food choices within these environments.

**Methods:**

Field studies are being conducted in primary schools, among children aged 9–12 years, in Greater Beirut, Lebanon and Greater Tunis, Tunisia. A stratified random sample of 50 primary schools (public and private) are selected and 50 children are randomly selected from grades 4-5-6 in each school. The study includes surveys with children, parents/caregivers, school directors, teachers, and nutrition/health educators to assess individual diets and the contextual factors that influence children’s food choices. Innovative locally adapted tools and methods such as game-based choice experiments, wearable cameras and neighborhood mapping are used to describe the environments in which children’s food choices are made.

**Discussion:**

The SCALE study will generate contextual knowledge on factors in school and neighborhood environments that influence child dietary behaviors and will inform multi-level interventions and policies to address childhood malnutrition (under-and over-nutrition). By integrating methods from various disciplines, including economics, data science, nutrition, and public health and by considering factors at various levels (home, school, and neighborhood), the study will identify levers for intervention with the potential to improve children’s dietary behaviors. This will help fill existing gaps in research on food systems and consequently guide positive change in Lebanon and Tunisia, with the potential for replicability in other contexts.

## Introduction

The last two decades have been marked by substantial dietary and lifestyle changes in the Arab region, where the prevalence of overweight and obesity among children is estimated to have doubled during this period [1–3]. In fact, diets have included ever-increasing shares of processed foods high in fats, sugar, refined grains and salt [4], together with low levels of physical activity [5, 6]. In middle-income countries of the Arab region, while stunting and micronutrient deficiencies persist, overweight prevalence estimates in children and adolescents are similar to those of high-income countries of the region, reaching about 30% in Lebanon and 20% in Tunisia [2]. Overweight in Arab adolescents has been linked to early metabolic syndrome, hypertension, cardiovascular diseases, diabetes, and musculoskeletal disorders; all conditions that are cause for higher proportions of disability-adjusted life years in the region [7]. Nearly a third of the region’s population is aged under 15 years [8], and if diet and overweight trends are not curbed, this youth bulge risks adding economic pressures on health systems, straining their capacity to deal with non-communicable disease (NCD) burdens.

Evidence suggests that children’s dietary habits are established early in life and continue into adolescence and beyond [9, 10]. Children’s weight status and food habits are influenced by many factors that span the socio-ecological model, including individual physiology and behaviors, family characteristics and interactions, as well as structural forces at the level of community, society, and policy environments [11–15]. Given that children spend a large proportion of their time at school, their eating patterns are also influenced by the food choices available in schools [16–20]. Beyond the confines of schools, aspects of the neighborhood environment, including accessibility to, amount and types of food outlets, as well as food marketing and advertising, are also associated with children’s food choices and body weight [21–24].

The ways in which obesogenic environments influence food choices have been shown to be context-specific [25, 26]. While there is considerable research from high-income countries [27, 28] and from studies that investigate the specific influences of external environments only [29], a better understanding of children’s immediate environments in a middle-income context is essential to inform interventions that address childhood dietary patterns, behaviors, food choices and overweight in the region.

Assessment of factors influencing food choices among children and adolescents is challenging, and is usually based on self-reported data, which are prone to measurement error including information and recall bias [30]. Technology-based tools enable an objective and comprehensive measurement of children’s nutrition-related behaviors and experiences around food [31–33]. As the popularity and use of mobile technologies and video gaming platforms increase, opportunities arise to use these tools to collect data on variables that affect food choices and diets. Such tools can also foster a ‘people-based’ approach to measuring these exposures [34]. These include factors in the child’s environment such as setting, social interactions and media exposure [32]. Digital technologies can give insight on children’s lived experiences and engage younger participants in documenting their own behavior, which may also lead to more accurate and representative data collection [30, 33, 35]. Such technologies have been positively received by younger participants and are feasible and acceptable [32, 33, 36, 37].

### Conceptual framework

**Fig 1:**
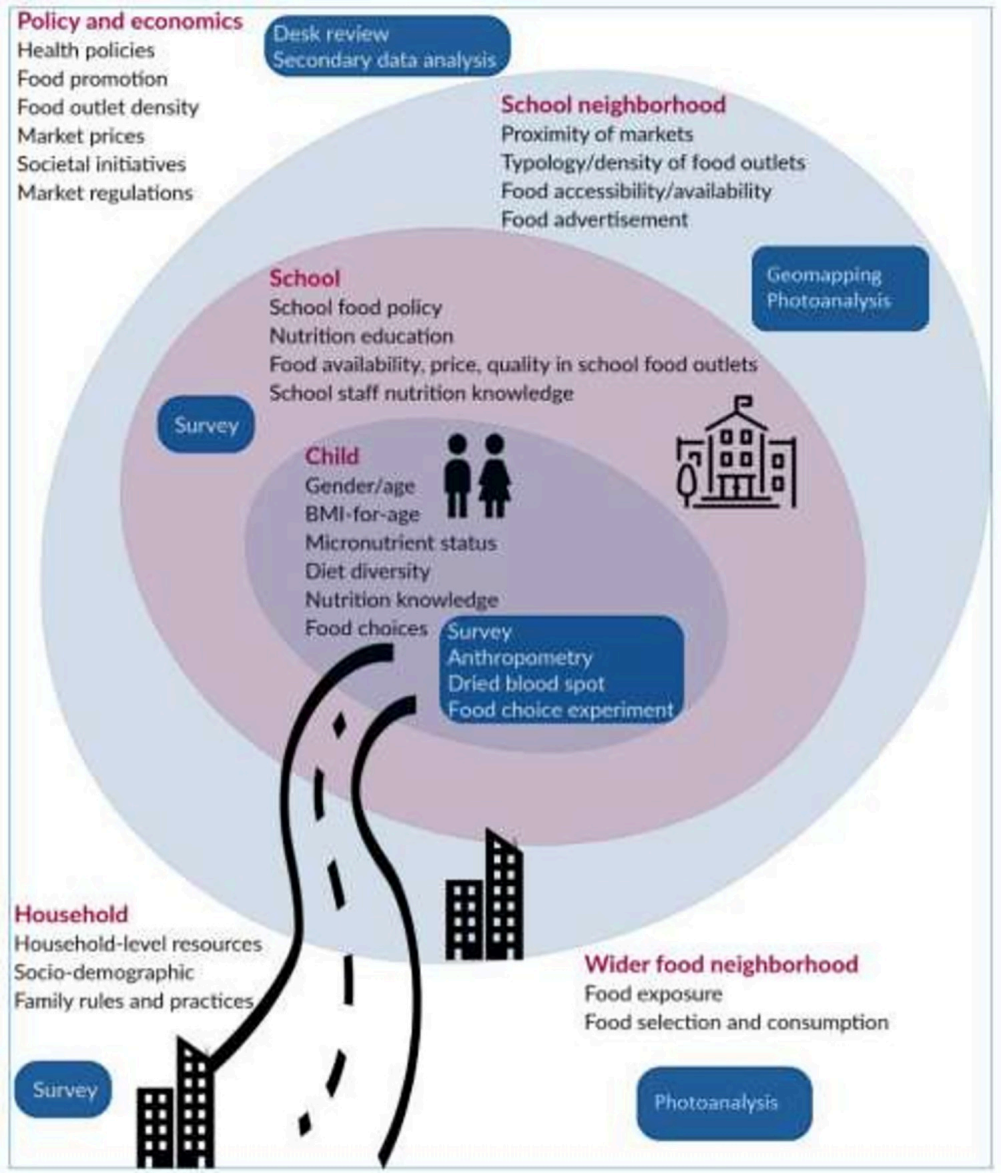
Ecological framework of the study. The figure shows the variables to be collected at each level and the methods to be used (blue boxes).

Fig 1 depicts the “**S**chool and **c**ommunity drivers of child diets in **A**rab cities; identifying **le**vers for intervention” (SCALE) study conceptual framework showing the levels of influence on children’s food choices, the variables to be collected by the SCALE study at each of these levels and the tools needed to collect each set of variables. The macro level includes the economic and policy environment. The meso level includes school food environments, the neighborhood environment of the school, and exposure to the wider neighborhood food environment in children’s trajectories to and from schools. The micro level includes the child’s household and individual behaviors and characteristics.

Macro-level factors such as food system policies have been recognized to improve diet quality, particularly among children and adolescents [38]. For younger children, the effects of macro-level and policy environments are mediated through their caregivers, whereas older children engage more directly with these environments [39]. The presence of, and proximity to, food markets that children and adolescents often encounter play a crucial role in defining children’s food choices, especially as these markets often sell non-nutritious foods with concerns about quality and safety [40]. Also, there are massive promotions of unhealthy snacks that are cheap, nutrient-poor and energy dense, and very limited investments to promote healthy foods [29, 41]. Additionally, nutritious foods recommended to children and adolescents can be expensive, particularly in low-income settings [42].

Intermediate (meso) structures such as school, neighborhood, and community level environments mediate the effects of societal forces on children’s eating behaviors by influencing exposures, perceptions, understandings, and behaviors.

Usually, children consume over a quarter of their daily caloric intake at school; as such, eating patterns in schools contribute significantly to diet quality, as well as subsequent health outcomes, including increased risk of obesity [16, 43–45]. With food availability being one of the most salient factors associated with food choice [43], the ready availability of nutrient-poor and energy-dense competitive foods [16, 43] at schools lead to increased energy intake and body mass index (BMI), and decreased fruit and vegetable consumption [16, 17, 46], with negative health implications. School food policies can modify the school environment and alter social norms relating to appropriate foods for children [47]. Food-related policies in schools, however, have not received enough attention in low and middle-income countries, and there is increasing financial dependence by schools on revenues generated by food sales [45], which are used to support food service and other school activities [16]. A better understanding of enablers and barriers to healthy eating behaviors in schools in such contexts is warranted to inform interventions to address childhood overweight [48].

Neighborhood and community-level environments represent another set of meso structures that influence children’s dietary intake beyond the confines of schools [49]. Reviews have reported a number of associations between, on the one hand, neighborhood environment characteristics–including the accessibility, amount, and types of food outlets–and, on the other hand, food choices and body weight among boys and girls [21, 22]. For example, living closer to convenience stores has been associated with an increased intake of unhealthy snacks, such as chocolate and crisps, among children [50, 51]. The price of food has also been associated with consumption [22], as documented in a study that found that a dollar increase in the price of fruits and vegetables decreased consumption by 6.3% [22, 52]. These environmental influences on food choice have been shown to be context-specific and to manifest differences in dietary intake [25, 53]. The complexity of food choice warrants further study of the contexts in which these choices are made [54]. Classifying both proximity and density of food outlets is a common method used to map spatial accessibility to food in different neighborhoods, and to assess associations between the food environment and individual behaviors [55]. Studies that have categorized food outlets and retailers and mapped them according to proximity to children’s homes and neighborhood density, have reported associations between these factors and food intake among children [51].

Being at the center of the framework, the micro level elaborates the child’s characteristics and autonomy in making their own food choices and decisions. Eating patterns in children and adolescents are influenced by several factors such as physical activity, taste preferences, and habits [40]. In addition, many other household-level factors shape children’s food choices such as food availability, accessibility and affordability, as socio-economic status can dictate food environments that children and their caregivers are exposed to [56]. Caregivers, play a key role in determining young children’s eating behaviors since they procure, prepare, cook and provide food [40].

This conceptual framework guides the design of the study in two urban centers of the Arab world: Greater Beirut, in Lebanon and Greater Tunis, in Tunisia. These two urban agglomerations provide examples of the nutritional shift occurring in many Arab countries. Lebanon and Tunisia are both middle-income countries that have experienced rapid rates of urbanization and economic development [1, 57] paralleled by a rapidly proceeding nutrition transition [3, 58] with overweight prevalence in children having long surpassed that of stunting [59]. This transition has typically been most rapid in urban contexts, where the consumption of starchy carbohydrates, food away from home, and processed foods including sugary snacks among children are on the rise [3, 60].

### Objectives

The SCALE study uses a cross-disciplinary approach and employs innovative locally relevant tools to assess school and community-level drivers of children’s dietary behaviors. The specific study objectives are to:

1. Develop innovative, context-specific methods that measure and account for the complex set of factors that play a role in children’s food choices and dietary behaviors
2. Describe the environments at the level of households, schools and communities in which children’s food choices are made
3. Identify points in the daily routine of children that represent threats to/opportunities for healthy eating
4. Quantify the impact of the obesogenic environment on schoolchildren’s food choices and their nutritional outcomes
5. Define context-specific multi-level interventions for influencing children’s food behaviors and diets related to the home, school and neighborhood environments

## Materials and methods

### Study area

In each country, the study focuses on the urban area around the capital city (Greater Beirut, in Lebanon and Greater Tunis, in Tunisia).

### Study design

We design this study to investigate and identify school and community drivers of children’s diets. We use mixed-methods to develop the innovative research tools. The tools are applied in a cross-sectional design to gather quantitative data on children’s individual, familial, school-level and neighborhood-level characteristics.

### Study participants

In both countries, the study population includes students enrolled in grades 4-5-6, in both public and private schools. The study also includes surveys with parents/caregivers, school directors, teachers, and nutrition/health educators.

Sample size calculations were based on simulations derived by Moineddin et al. [61] to reduce relative bias of estimates in multi-level logistic regression models. The recommended minimum group size was found to be 50 with at least 50 observations per group for the derivation of valid estimates. The assumptions underlying this recommendation were verified for several key study indicators using data from published literature (e.g. expected prevalence and intra-class correlation coefficients for diet diversity categories and overweight/obesity within schools) [62, 63].

We therefore use a two-stage sampling approach to recruit a representative sample of 8 to 12-year-old schoolchildren. A random sample of 50 schools, stratified by school type (public and private), is selected from the list of schools provided by the respective Ministries of Education in both countries. Accounting for the expected non-response rates, the team contacts the schools to schedule a meeting with the directors to explain the study objectives, protocol, and timeline. From the schools that agree to participate in the study, we randomly select students from grades 4-5-6 and send parental consent forms home with the selected students, with the aim of enrolling 50 students per school: for a total of 2,500 students per city.

In the schools that agree to participate, we invite the school/section director, teachers and nutrition/health educators that teach grades 4, 5 and 6 to participate in the study. We recruit one director, 5–10 teachers, and 1–3 nutrition/health educators present at school on the day of data collection to participate in the study.

### Study procedures

This study protocol employs various research methods which address different levels of the socio-ecological model. Based on the aforementioned conceptual framework (Fig 1) we present hereafter the details of the different tools employed at each level in the study.

## 1. Macro and meso levels

This study aims to map structures at the macro and meso levels; that is the economic and policy environment along with school food environments, the school neighborhood environments, and exposures to the wider neighborhood food environment in children’s trajectories, respectively. These structures are mapped using four main methods.

### 1.1 Desk review

A desk review of the broader food-related economic and policy environment at the country-level for Tunisia and Lebanon is performed. This provides comparable information on the macro-level context within which intermediate structures (schools and neighborhoods) exist and operate in each country. For this, we systematically collect and analyze published articles, available reports and policy documents describing the policies and economic, social and political factors that influence child diets and nutritional status in Tunisia and Lebanon; we include sources specific to each country, as well as more general sources on the region if relevant. Documentation pertaining to existing policies and initiatives established to reduce childhood overweight in Lebanon and Tunisia are also compiled. Other complementary sources are collected to document economic and agricultural policies of the country, including those that influence exposure to food promotion (exposure to food advertisements, purchase prompts at food outlets, food labeling and packaging), food outlet density (supermarket density, fast food outlet density), market prices (imported vs locally produced goods, food subsidies), societal initiatives related to child nutrition, social norms related to household decision making around food choices, and other market regulations and policies affecting food choices.

### 1.2 School survey

We conduct a school survey, administered to school principals and teachers in order to effectively profile school food environments and assess children’s exposures to different components of this environment. This survey builds on standardized tools to examine school food-related policies and compliance with these [64], the extent of nutrition education available in the curriculum, knowledge and attitudes of teachers towards nutrition and food, food availability, as well as price and quality in school food outlets [45, 65, 66]. This survey consequently generates two sets of data: first, school profiles that can be used to inform advocacy and intervention development; second, a list of school-level variables to include in multi-level analyses of the determinants of children’s food choice and diets.

### 1.3 Wider neighborhood food environment and trajectory mapping

To capture exposure to different factors in children’s food environments, we use an innovative set of tools that build on simple digital technologies to collect comparable data that can be quantified and analyzed across the two countries. We adopted a user-centered design approach to develop an innovative tool using wearable cameras with the aim of capturing neighborhood food environments and food trajectories of children. Focus groups and design activities were conducted with parents, children and school staff. These data informed the design of this component in an ethical and culturally acceptable manner with the detailed methods described elsewhere [67]. Based on ethical frameworks [31, 68, 69] and findings from the formative research phase, we developed a comprehensive camera model and data collection protocol detailed in Zoughby [70]. This consists of a wearable camera, attached to a strap worn over the child’s clothes, that automatically captures continuous footage of what children are exposed to in their daily trajectories to and from school. Only children in grade 6 are invited to participate in this component. Those whose parents agree to participate, wear the camera for one full day, outside the confines of school, mainly on the road/trajectory to and from school, and inside their homes (if parental consent and child assent is obtained). Timestamped images are extracted from the recorded footage and consequently filtered using machine learning models to only retain the images relevant to the study; those related to food advertisement, food outlets, food purchasing and food/beverage consumption and to blur all faces in the images, therefore generating a dataset of pseudonymized images related to food exposures. These are then further classified according to the NOVA classification–a classification that categorizes foods into four groups according to the extent of food processing [71]. These data will allow us to uncover and quantify the magnitude and the quality of exposures and cues to food choices and eating behaviors in children’s daily trajectories.

### 1.4 School neighborhood food environment mapping

We also map the community food environments that children are exposed to, including food outlets’ proximity to schools, typology, and density, and child exposure to food advertisement in the neighborhoods of the selected schools [51, 72, 73] A data collection module is designed to identify, enumerate, classify, and map food stores/advertisements within an 800-meter road network buffer around selected schools [73]. This is programmed on a mobile phone using two applications: Collector Classic and Survey123 (ESRI Inc., Redlands, CA). Data collectors are given the programmed mobile phones and instructed to (a) walk around the school according to a planned route using Collector Classic, and (b) collect data on food outlets and food advertisements using Survey123. The geographic coordinates of each food outlet/food advertisement, and a geotagged picture of the outlet/advertisement are collected. These are categorized into typologies of outlets (e.g. supermarket, ambulant vendor, pastry shop) as well as food groups for advertisements (e.g. fresh fruits and vegetables, processed meats, sugar-sweetened beverages). Then, they are all further classified according to the NOVA classification [71]. The geocoded locations of schools, and detailed food outlet and food advertisement classifications are then projected to GIS software in order to analyze the proximity, density, and clustering of food stores and advertisements relative to schools (ArcGIS 10, ESRI Inc. CA). This method is described in detail elsewhere [74].

## 2. Micro level

The macro and meso-level data collected are complemented by structured interviews with schoolchildren and parents, and anthropometric measurements and micronutrient assessment of schoolchildren. For this, two methods are used allowing us to explore correlations between behavioral and nutritional variables as well as factors in the school and neighborhood environments that may influence them. By doing so, we generate individual-level variables, including socio-demographic status, dietary behaviors, food choices, nutritional outcomes, and sedentary behaviors [75, 76].

### 2.1 Parents

Family/household-level indicators are collected from children’s parents/caregivers through a 15-20-minute telephone interview using a structured questionnaire programmed on tablets. This questionnaire includes general socio-demographic family and household characteristics (age, sex, education, employment, household size, assets, expenditures, wealth). The questionnaire also includes a section on family food-related habits and food-related parenting style and modeling [77–79]. We collect parents’ perceptions of the home food environment [80, 81] and administer the Arab Family Food Security Scale (AFFSS), previously validated in Lebanon [82, 83]. The household diet diversity scale is additionally used as a proxy of household access to a variety of foods [84]. We lastly assess parents’ level of nutrition knowledge using eight questions adapted from the General Nutrition Knowledge Questionnaire (GNKQ) [85] and collect a self-report of parental anthropometric measurements.

### 2.2 Children

To collect child-level outcomes and co-variates, we employ four tools. First, students in grades 4-5-6 participate in a structured face-to-face interview and answer the children’s questionnaire. The tablet-programmed-questionnaire lasts 30 minutes and includes questions on demographic variables (age and gender), food consumption and food purchase habits in and around school [86–92]. It also includes a module on child diet diversity, which is used as a proxy for children’s diet quality and nutrient adequacy [84]. We further collect data on children’s physical activity levels and sedentary behaviors [93, 94] as well as assessing children’s level of nutrition knowledge. Second, trained data collectors record students’ anthropometric measurements, including height, weight (to be able to calculate BMI for age) and waist circumference (WC). These measurements are taken individually, in a private space using a standard stadiometer for height, a calibrated weighing scale for weight, and a measurement tape for WC. Third, a registered nurse conducts a finger stick to obtain around 400 μL of blood, part of which is used to measure hemoglobin on the spot using a portable hematofluorometer (hemocue) to gage anemia, and the other for further micronutrient analyses [95]. Serum aliquots (between 50–100 μl each) are shipped to a laboratory in Germany, where a single ELISA has been developed to measure plasma ferritin, soluble transferrin receptor, retinol binding protein (RBP), C-reactive protein (CRP), and Alpha-1-acid glycoprotein (AGP) [95] allowing us to establish children’s iron and vitamin A status.

**Fig 2:**
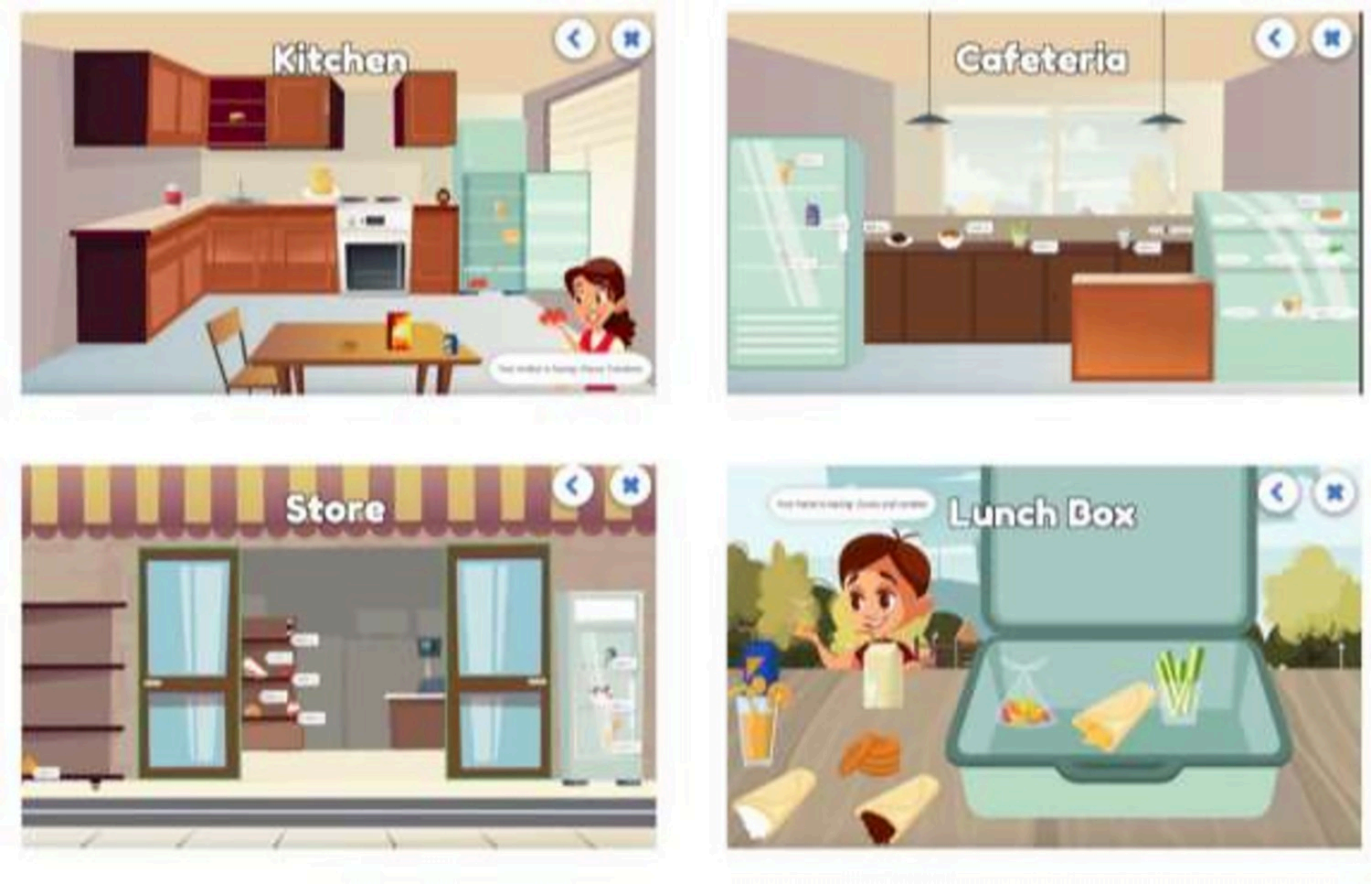
Examples of different scenarios and choice tasks from the choice experiment.

The last tool used is a digital game that employs a series of choice experiments designed to vary key factors that shape children’s food choices [96–99]. The choice experiments are tailored to the context of schools and homes in Lebanon and Tunisia and present the most frequently consumed foods by schoolchildren based on previous literature. We worked with a software developer and a graphic designer to ensure a modular, customizable and appealing game (Fig 2). The choice experiment is composed of six different contexts or meal types within which children are asked to select hypothetical food choices: (1) breakfast, (2) road to school, (3) recess, (4) lunch, (5) snack and (6) dinner. In each section, children are presented with repeated choice tasks, each composed of nine items: three beverages, three side meals and three meals. Children get to choose their preferred items based on items’ attributes and contextual effects that apply. Attributes that are being explored are item placement (accessible or hidden in a cupboard), preparation (ready to eat or requires an additional step to prepare), and price, when applicable. Contextual effects are applied to the choice tasks in general and hence equally to all items within this task (e.g. supervision by parents, food choices of friends and peers). Also, the level of healthiness of each item is studied implicitly. Each of the three items representing a beverage, side meal or meal, was selected to the choice task from an exhaustive menu of ‘healthy’, ‘medium healthy’ and ‘unhealthy’ articles, respectively, that are typically present in the Lebanese or Tunisian diet. The econometric analysis [100] of these choices enables the estimation of preference intensities for each food item as inflected by the type of food/beverage articles, its attributes and the contextual effects under which it is presented.

### Analytical approach

**Fig 3:**
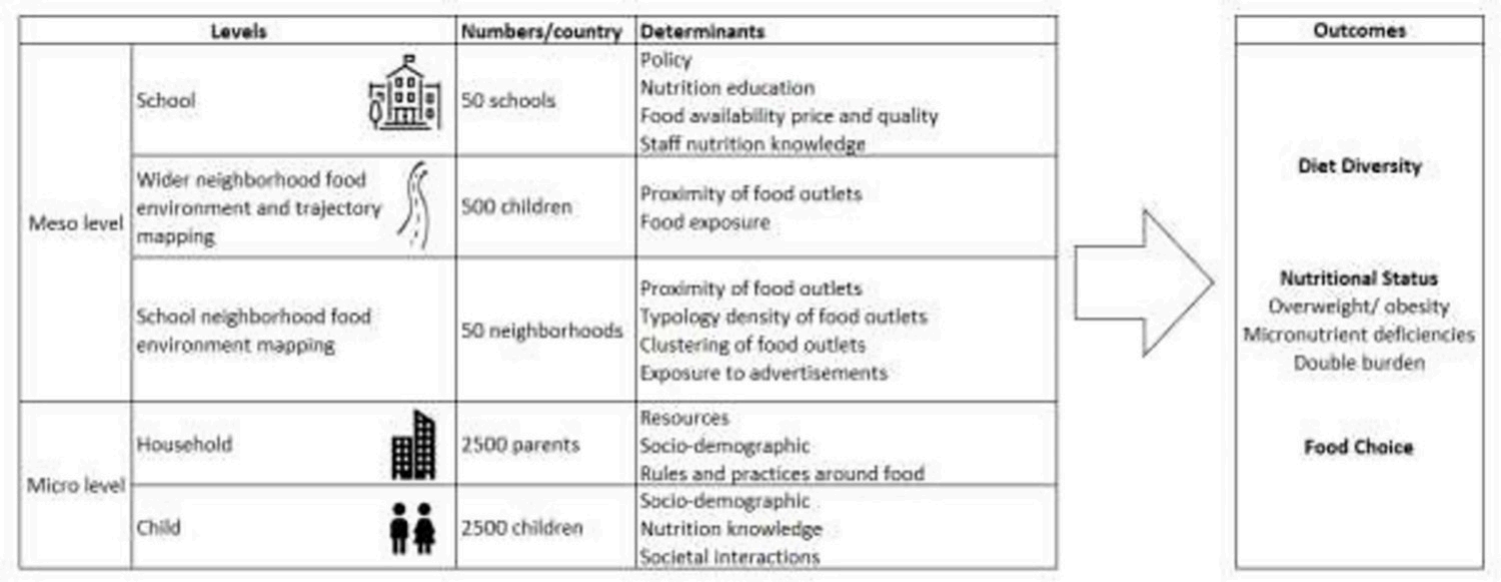
Meso and micro level factors that influence children’s diet diversity, nutritional status, and food choice.

Data collected from the multiple levels will be merged and analyzed using standard statistical methods. Fig 3 illustrates the set of expected outcomes per phase and analysis level.

Descriptive statistics will be used to describe child diets and socio-demographic variables at the child and household level. School-level variables will be generated in regard to policy, nutrition education and food availability patterns. At the school neighborhood-level, data will be used to generate variables related to proximity, density, and typology of food outlets. For the wider neighborhood-level, we will use deep learning models to conduct photo analysis and quantify the intensity, frequency, types and healthiness of food exposures captured by wearable cameras, stratified by gender, socio-economic status, geographic areas, and nutritional status.

The determinants of children’s food choices, diet quality, and nutritional status will be examined using logistic and linear regression models. Multilevel models [101, 102] will be used to analyze the various levels of influence on child diets and nutritional status, allowing for the analysis of hierarchical data [103–106]. Neighborhood and school environmental factors, individual socio-economic characteristics and contextual effects in choice tasks, will be accommodated by the econometric analysis as preference intensity shifters. We will take a comparative approach to analyze the similarities and differences across the two study sites, as well as across neighborhood typologies, school typologies, socio-economic strata, and gender. All statistical analyses will be conducted using STATA version 15 (STATA Corporation, College Station, Texas, USA).

The multiple layers of analysis included in this project will ensure the triangulation of data from different sources and will allow us to fill knowledge gaps that exist at each of the levels of analysis. The documentation of children’s trajectories informs data collection at every level and allows for the validation of data collected from child and parent reports. The photo-documentation of trajectories can also be used to illustrate salient findings in the dissemination of study results.

### Ethics approval and consent to participate

All applicable institutional and governmental regulations on the ethical use of human volunteers were respected. In Lebanon, the study has been approved by the American University of Beirut (AUB) Institutional Review Board (IRB) on December 23, 2019 (IRB ID: SBS-2019-0306). In Tunisia, the survey protocol was reviewed and approved by the Ethics Committee on Human Research of the National Institute of Nutrition and Food Technology (INNTA) (Visa n° 03/2019) on July 12, 2019 and by the National Council of Statistics in Tunisia (Visa n° 06/2019) on April 28, 2019. All parents/caregivers gave their written informed consent.

The information we collect herein is treated with the utmost confidentiality; every reasonable effort is made to keep the records confidential and preserve participants’ anonymity across the various study components. Consent for participation is to be sought from all participants either in oral or written form, both documented in the records. All participation is voluntary; participants are free to opt-out of any component of the study including the fingerstick and wearable camera components.

### Current study status and timeline

Data collection began in Tunisia in January 2020 and was completed in November 2020. As for Beirut, data collection is set to commence in September 2021.

#### Tunisia

Data collection began on January 30^th^, 2020 with the implementation of child surveys, anthropometry, biomarker collection, school environment survey, and the wearable cameras protocol. However, the emergent COVID-19 pandemic, which resulted in school closures, forced the team to halt data collection on March 11^th^, 2020. The team completed 29 out of the 50 schools at the individual, family, and school level. Following this, extensive field work was conducted to collect the neighborhood mapping data. School-level data collection resumed on October 12, 2020 under stringent COVID-19 precaution measures. The second wave was slower because of the adopted hybrid system in schools and infection mitigation protocol put in place in the country. Data collection was conducted on two consecutive days for each school in turn allowing us to reach the desired sample size on November 16, 2020, despite the difficult circumstances. In parallel, phone interviews with parents were conducted.

#### Lebanon

As for Lebanon, data collection was planned to commence in October 2019. However, as a result of worsening economic conditions, social movements led to road and school closures in October-November 2019 and postponement of fieldwork to 2020. The emergent COVID-19 pandemic then shuttered schools from March 2020 with schools in Lebanon only opening for a total of four non-consecutive weeks of hybrid schooling thus far in the academic year 2020–2021. Beirut was also exposed to one of the largest non-nuclear explosions in history in August 2020 causing death, injury, destruction and further economic hardship. These overlaps between the economic, COVID-19 and Beirut blast crises have contributed to an increase in poverty and food insecurity, changes in markets including the food environment, and an overall increase in vulnerability of residents of Greater Beirut. It will remain essential to conduct this study when schools reopen in the aftermath of these crises; data collected will capture the new reality of this population and allow us to identify key points of intervention to improve children’s diets—essential to “building back better” post crises.

## Discussion

In the context of the rapid nutrition transition experienced by middle-income countries of the Arab region, children’s food choices and dietary behaviors affect growth and development and are early risk factors for the development of NCDs. The current research base on overweight among children in the Arab region has considerable gaps in how it has been problematized, often limited to assessment of individual-level risk factors [107, 108]. In order to inform policy and community-based interventions, there is a need to measure factors beyond the individual that shape children’s exposure to the food environment, their food choices and subsequent health outcomes. To our knowledge, this is the first study that takes an ecological approach to build a robust evidence based on school and neighborhood-level food systems and assess their influence on food choices and behaviors among children in the Arab region.

For this, we have developed innovative and contextually validated tools and methods to inform effective solutions to unhealthy diets in children. By mapping children’s food-related trajectories to and from schools and assessing the influence of these trajectories and exposures on children’s dietary behaviors, the study takes a systems approach to generate an in-depth understanding of the drivers of children’s diets in two middle-income countries experiencing a nutrition transition.

This investigation of how factors at the individual, family, and neighborhood levels influence food behaviors and practices among boys and girls will provide an evidence base for efforts targeting the rising prevalence of unhealthy diets and overweight, which has implications for growth and development in the short to medium term and NCD prevention in the long-term. The knowledge generated from this research will ultimately present practical solutions at different levels (policy, school, advertisement, and neighborhood), that enable and consequently promote mechanisms that have the potential to change dietary behaviors among children. The findings of this study will further inform culturally appropriate and context-specific interventions which could be relevant to the broader Arab world; including urban low-income countries that are experiencing similar rapid epidemiological and nutrition transitions.
